# Chronic pain syndromes: overlapping phenotypes with common mechanisms

**DOI:** 10.12688/f1000research.16814.1

**Published:** 2019-03-05

**Authors:** Geoffrey Owen Littlejohn, Emma Guymer

**Affiliations:** 1 Department of Medicine, Monash University, Clayton, Victoria, 3168, Australia; 2 Department of Rheumatology, Monash Health, Monash Medical Centre, Clayton, Victoria, 3168, Australia

**Keywords:** fibromyalgia, regional pain syndrome, complex regional pain syndrome, central sensitisation, criteria, mechanisms

## Abstract

The common chronic pain syndromes of fibromyalgia, regional pain syndrome, and complex regional pain syndrome have been made to appear separate because they have been historically described by different groups and with different criteria, but they are really phenotypically accented expressions of the same processes triggered by emotional distress and filtered or modified by genetics, psychology, and local physical factors.

## Introduction

Understanding of the high-impact chronic musculoskeletal pain conditions of fibromyalgia (FM), regional pain syndrome (RPS) and complex regional pain syndrome (CRPS) continues to grow ^1– 3^. Each of these conditions has a characteristic clinical phenotype with reproducible and distinguishing clinical features that permit establishment of criteria in order to classify the condition for research purposes or to diagnose the condition for clinical management purposes. Here, we present evolving information on these disorders and propose areas of further enquiry.

## Discussion of recent literature

In regard to FM, a number of such criteria have evolved on the basis of the essential characteristics of the condition ^4^. The association between widespread pain and widespread abnormal tenderness was the key contributor to the 1990 American College of Rheumatology (ACR) Classification Criteria, established for research into this condition ^5^. In 2010 and again in 2011, the criteria were modified to accommodate other typical features by adding scores for sleep disturbance, fatigue, cognitive dysfunction, headache, abdominal pain, and depression to a numerical self-report of the number of painful or tender regions in the body ^6, 7^. Thus, a mix of clinical features pertaining to both central and peripheral components of the pain-related neural systems was included. Disorders characterised by regional pain are accommodated by these criteria if the symptom ratings are high ^4, 7^. These criteria were designed for both classification and diagnostic purposes. They were further modified in 2016 in order to focus on widespread pain rather than regional pain, but the essential elements remained. Each of these criteria is deemed valid for classification or diagnosis of FM (or both) ^8^.

CRPS criteria have also evolved over time with the focus being on identifying characteristic peripheral clinical features rather than including any of the “central” ones, as is done in the FM criteria. The most commonly used criteria iterations are the so-called Budapest Criteria ^9^. Variations in scoring the components of these criteria permit use in classification and research and also for clinical diagnosis. Thus, the criteria reflect key elements of the CRPS clinical phenotype which include variable levels of regionalised allodynia (abnormal sensitivity to non-noxious stimulation, such as light touch), colour or temperature change in the involved region, altered growth of hair or nails, abnormal motor function, and changes in sweating and swelling in the region. These features all occur in the context of pain felt to be inappropriately high relevant to the initial triggering or provocative stimulus. In the case of both FM and CRPS, there must be no other clinical cause that could account for these symptoms ^8, 9^.

RPS is characterised by segmental pain and allodynia occurring in the absence of a defined neurological or musculo-ligamentous cause and usually presents adjacent to a spinal structure, particularly the low cervical, mid-thoracic or low lumbar area ^3^. This condition has high levels of muscular tightness within the region as well as non-neuroanatomical dysaesthesia (abnormal cutaneous sensations). Apart from the ACR 2010/2011 criteria, where the diagnosis of FM may be applied, there are no widely accepted validated criteria for this common chronic pain condition ^3^. RPS is often triggered by injury to a proximal musculoskeletal structure and is often described in lay terms, such as whiplash, repetitive strain syndrome or non-specific back pain ^3^.

Although the clinical phenotypes of FM, RPS and CRPS differ, the conditions are linked through the dominant clinical features of pain, fatigue, cognitive dysfunction, poor sleep, allodynia, swelling, dysaesthesia and cutaneous circulatory changes ^3, 10^. These individual characteristics each occur on a spectrum ranging from mild to severe. This is reflected in the overall presentation of each particular phenotype which may also range from minor to severe. Many people move between different levels of symptoms, suffering from flare-ups of symptoms or remission of symptoms.

Indeed, in FM, the current criteria can be used to rate any one individual on a scale of increasing tendency to reach the diagnosis of FM, termed fibromyalgianess ^8^. Increasing scores on the scale, even if in an FM sub-diagnostic range, will associate with poorer pain-related outcomes in a number of interventions ^11– 13^. Additionally, patients with either RPS or CRPS may transition to more widespread pain and fulfil criteria for FM ^10, 14^.

Thus, FM, RPS and CRPS are related and as a group fall into the paradigm of chronic overlapping pain conditions ^15^. As such, they each also associate with other disorders in this group, which might include irritable bowel or bladder syndrome, myalgic encephalomyelitis/chronic fatigue syndrome, restless legs syndrome and migraine, among others ^15, 16^. These conditions are classified as “chronic primary pain” in the classification system proposed by the International Association for the Study of Pain, developed in conjunction with the World Health Organization, for use in the International Classification of Diseases, 11th edition ^17, 18^.

A key pathophysiological process behind each of these syndromes is central sensitisation. This term denotes increased response to a given stimulus in a sensory system through changes in central nervous system functioning ^19, 20^. Most commonly, this does not involve any peripheral sensory input to initiate the process. In the conditions under discussion, all characterised by significant pain, central sensitisation involves a wide range of changes in sensitivity within the pain-related neural systems. In FM and CRPS, changes in sensitivity have been identified at several levels in the pain-related neural systems involving both central and peripheral nervous systems ^10, 21^.

A number of alterations in neural functions favouring a “top-down” mechanism contributing to central sensitisation have been identified in FM. Changes within the brain networks related to sensory processing are seen within the central nervous system in FM ^21, 22^. For instance, changes in connectivity between the insular and the default mode network correlate with pain levels in FM ^21, 23, 24^. There is also altered control of descending pain modulatory pathways in FM ^21, 25^, a situation that will change dorsal horn sensitivity, response and subsequent transmission of incoming sensory information that relates to pain. Finally, there are also changes in the peripheral nervous system characterised by neurogenic inflammation in the skin and muscles ^10, 26^. Similar changes are likely to be evident in the dorsal horn of the spinal cord and, through increased activity of glial cells, may be present in the brain ^21, 26^.

The key peripheral neural fibre types that are involved in symptoms that associate with FM, RPS and CRPS are the large-fibre mechanoreceptors. These fibres usually provide innocuous mechanospacial information to the brain subconsciously. Where there is central sensitisation, information derived from these fibre types, coming from peripheral tissues, “gains access” to the pain system through pathways in the deep dorsal horn, which allows the translation of otherwise innocuous sensory information to be registered as a pain sensation in the brain. In this setting, low-level activity or sustained postures will induce pain, a characteristic of each of these pain disorders.

All of the changes described above are likely to be present in CRPS, although there is much more information in FM and RPS in regard to central neurophysiological changes ^27, 28^. In CRPS, a large amount of work has occurred in the periphery and this does show high levels of neurogenic inflammation in the periphery, particularly in the early stages of the condition ^10, 29^. Fewer studies have been performed in RPS, but because of the similar clinical characteristics of the main presenting features, it is likely that central sensitisation with related neurogenic inflammation is present in that condition as well ^28^.

The mechanisms behind the distribution of the pain in each of the different clinical phenotypes are unclear. However, in RPS, the ubiquitous presence of segmental symptoms and signs adjacent to a spinal region suggests that certain biomechanical factors are important. These likely include the participation of the additional mechanism of referred pain from deeply placed structures. This would be explained by mechanoreceptor input from deep spinal musculo-ligamentous structures, perhaps associated with strain or degenerative change, that interacts with sensitised dorsal horn neurones at the relevant spinal level. This mechanism might also come into play in the widespread pain of FM. In contrast, in CRPS, the pain is, at least initially, characteristically peripheral and regional and more intense and accompanied by significantly heightened neurogenic inflammation compared with FM or RPS. CRPS is usually triggered by peripheral tissue damage which appears highly relevant to inducing the localised intense sensitisation process that follows. As time passes, however, most patients with CRPS develop a pain pattern that involves the whole of the involved limb with features of RPS and later often becomes more widespread, fulfilling criteria for FM.

In each of these pain phenotypes, it is also common to find increased sensitivity within non-pain neural systems. This can include light sensitivity, noise sensitivity, bowel or bladder sensitivity, postural hypotension, dizziness, tinnitus, and other apparently difficult-to-explain symptoms. This implies that the sensitivity changes are broader than those that relate only to pain-related neural mechanisms.

The question remains as to the cause of the cascade of events that lead to the increased sensitivity which is present in a variety of neural systems. In each of FM, RPS and CRPS, clinical observation indicates the critical occurrence of stress in the patient’s background and, more importantly, the presence of emotional distress. That is, the patient manifests a clinically relevant emotional response to the situation in which they find themselves. This in turn involves a complex mix on inputs from societal, personal and psychological sources.

The ensuing emotional distress likely invokes ancient pre-programmed patterns of neural algorithms that initiate the stress response and activate related changes in neural sensitivity. This response occurs on a spectrum and interacts with peripheral neural systems that are primed by local biomechanical factors. The resultant clinical phenotypes of FM, RPS and CRPS thus also occur on a spectrum modulated principally by top-down neural process but also influenced by a number of local biodynamic factors.

## Conclusions

Although significant understanding of the general descriptions and pathophysiological processes of these syndromes has been achieved as has been indicated in this review, there is still a lot of detail to fill in.
